# World’s Purity Federation

**Published:** 1915-03

**Authors:** 


### World's Purity Federation.

What has been said in the two preceding editorials is well in point with any movement tending to mould social customs. What becomes of general use by one generation, whether beneficial or harmful, is reflected in the next through social heredity. And unless this influence is of a moral nature the change will be retrogressive, as the test for fitness to live is the moral standard. To survive requires not only physical fitness but ethically best.

The World's Purity Federation is one of those agencies that is actively at work in seeking to better social conditions and consequently moulding the social heritable units of the future. It earnestly desires to call attention to the Ninth International Purity Congress which meets in San Francisco July 18 to 24. Its purpose is, by public sentiment and co-operation, to eradicate the white slave traffic and public vice in the world and the promotion of true purity. By a large delegation of serious minded men and women at this congress it expects to create a universal sentiment that will have dynamic force. This is true philanthropy and the very best practical eugenics, and it deserves the earnest support of every man and woman in the country.

Information can be secured by writing the president, B. S. Steadwell, La Crosse, Wis. Do it now.

